# An approach to quantum-computational hydrologic inverse analysis

**DOI:** 10.1038/s41598-018-25206-0

**Published:** 2018-05-02

**Authors:** Daniel O’Malley

**Affiliations:** Computational Earth Science Group, Los Alamos National Laboratory, Los Alamos, NM 87507 USA

## Abstract

Making predictions about flow and transport in an aquifer requires knowledge of the heterogeneous properties of the aquifer such as permeability. Computational methods for inverse analysis are commonly used to infer these properties from quantities that are more readily observable such as hydraulic head. We present a method for computational inverse analysis that utilizes a type of quantum computer called a quantum annealer. While quantum computing is in an early stage compared to classical computing, we demonstrate that it is sufficiently developed that it can be used to solve certain subsurface flow problems. We utilize a D-Wave 2X quantum annealer to solve 1D and 2D hydrologic inverse problems that, while small by modern standards, are similar in size and sometimes larger than hydrologic inverse problems that were solved with early classical computers. Our results and the rapid progress being made with quantum computing hardware indicate that the era of quantum-computational hydrology may not be too far in the future.

## Introduction

Classical computers have had a dramatic impact on hydrology for decades^1–9^, due largely to the exponential growth in computing power predicted by Moore’s law^10^. However, this growth is not expected to continue indefinitely and has already begun to slow^11^. Quantum computing is an emerging alternative to classical computing. There are different approaches to quantum computing including quantum gate arrays^12^ and quantum annealing^13^. Currently, gate-based quantum computers are composed of *O*(10) bits while quantum annealers have *O*(10^3^) bits, rendering quantum annealers more suitable for our purposes. We note that the situation regarding quantum computing hardware is evolving rapidly^14^. Here, we explore the use of a D-Wave 2X quantum annealer to solve several hydrologic inverse problems.

This work can be seen as an early step toward quantum-computational hydrology. As such, we find it appropriate to compare to early work from classical computational hydrology. This may be surprising, since quantum computing is popularly viewed as a technology that will instantly render classical computing obsolete. The reality is that not only have have classical computers made incredible improvements over the past several decades, but methods in computational hydrology have also seen significant improvement. Before quantum computers are of practical use for hydrologic inverse analysis, it is likely that there will be improvements in both the methods presented here and quantum computing hardware.

Inverse methods in hydrology can be broadly categorized as being either direct or indirect^1^. Indirect methods require repeated model runs (e.g., predicting hydraulic heads in an aquifer) and generally proceed by improving the model parameters (e.g., permeabilities, specific storage, etc.) in an iterative process until a convergence criteria is met (e.g., model predictions match observations within a given tolerance). Direct methods do not require repeated model runs and are generally less computationally demanding. However, they typically require the hydraulic head to be known throughout the aquifer. Because of their light computational demands, direct methods were commonly used in early computational hydrology^2,15,16^ and are still in use today^17^. Indirect methods are now much more common, primarily because they do not require the hydraulic head to be known throughout the aquifer.

The D-Wave 2X imposes onerous computational restrictions which make direct methods a natural first step. The approach we take is similar to some early hydrologic inverse analyses (e.g., the work of Hefez *et al*.^2^) where inverse problems were formulated as linear and quadratic programs^18^. Quadratic programming versions of the inverse problem were found to be superior to the linear formulation, but were difficult to solve because tools for solving quadratic programs where not readily available^2^. The D-Wave 2X deals with binary quadratic programming problems natively, and we use an approach here that formulates a hydrologic inverse problem as a binary quadratic program. The problems solved here with D-Wave’s third generation chip are small by modern standards, but they are larger than the problems solved by Hefez *et al*.^2^ which used a similar methodology and was published between the release of Intel’s third and fourth generation chips.

The remainder of this manuscript is organized as follows. First, the methods that were used to solve the hydrologic inverse problems via quantum annealing are presented. The focus of the methods is largely on the process of transforming a graph associated with the quantum annealer into a graph associated with the hydrologic inverse problem, a process called embedding. Next, we examine three examples of hydrologic inverse problems that were solved with a D-Wave 2X quantum annealer. These three examples include a small 1D problem for illustrative purposes, a larger 1D problem, and a 2D problem. Finally, we discuss the results and indicate next steps.

## Methods

Here we describe how to solve a direct inverse problem for steady-state 1D and 2D aquifers as a binary quadratic program. The underlying mathematical equation is ∇⋅(*k*∇*h*) = 0 where *h* is the head and *k* is proportional to the permeability (hereafter, we will refer to *k* as the permeability). We treat the problem in a non-dimensional setting, though any consistent dimensionalization could be used. We will use a finite difference discretization and assume for the direct inverse problem that *h* is known at all grid points. The inverse problem is to estimate *k* throughout the domain. A binary decomposition of *k* will be used: *k*_*i*_ = *k*_*l*_ + *q*_*i*_(*k*_*h*_ − *k*_*l*_) where *k*_*i*_ denotes the value of *k* at some node in the domain, *k*_*l*_ is a “low” permeability value, *k*_*h*_ is a “high” permeability value, and *q*_*i*_ is an unknown binary variable that must be inferred. Note that if *q*_*i*_ = 0 then *k*_*i*_ = *k*_*l*_ (i.e., the permeability at node *i* is low), and if *q*_*i*_ = 1 then *k*_*i*_ = *k*_*h*_ (i.e., the permeability at node *i* is high). This approach allows us to segment the domain into a high permeability region and a low permeability region, similarly to level set methods^19^.

In one dimension, the finite difference approximation at head node *i* is (assuming Δ*x* = 1)

1 $$0={k}_{i-1}({h}_{i-1}-{h}_{i})+{k}_{i}({h}_{i+1}-{h}_{i})$$

where *k*_*i*_ is the permeability between head nodes *i* and *i* + 1. Squaring the right hand side of Eq. 1, substituting *k*_*i*_ = *k*_*l*_ + *q*_*i*_(*k*_*h*_ − *k*_*l*_), summing over *i*, and neglecting a constant term that does not depend on any of the *q*_*i*_’s, we obtain a quadratic function

2 $$f({\bf{q}})=\sum _{i=1}^{n}{a}_{i}{q}_{i}+\sum _{i=1}^{n-1}{b}_{i}{q}_{i}{q}_{i+1}$$

where there are *n* + 1 head nodes in the domain, and the *a*_*i*_’s and *b*_*i*_’s (which depend on the *h*_*i*_’s, *k*_*l*_, and *k*_*h*_) can be readily assembled by a classical computer. It is important to note that the coefficient of *q*_*i*_*q*_*j*_ is zero unless |*i* − *j*| = 1. In the jargon of D-Wave programming, we say that *q*_*i*_ is “coupled” to *q*_*i*−1_ and *q*_*i*+1_, but is not coupled to any other bits. If we phrase this in terms of the permeabilities rather than the bits, we would say that *k*_*i*_ is coupled to *k*_*i*−1_ and *k*_*i*+1_. The fact that there are so few quadratic terms makes it possible to solve problems where the number of *q*_*i*_’s is almost as high as the number of bits on the D-Wave chip (each bit on the chip is only coupled to a few other bits). We also point out that there are no terms like ${q}_{i}^{2}$, because ${q}_{i}={q}_{i}^{2}$ when *q*_*i*_ is binary. Note that when $f({\bf{q}})$ = 0, Eq. 1 is satisfied for all *i*, and the equation is approximately satisfied when $f({\bf{q}})$ is small. Because of this, the function $f({\bf{q}})$ is the objective function that will be minimized to estimate the permeability throughout the domain.

**Figure 1:**
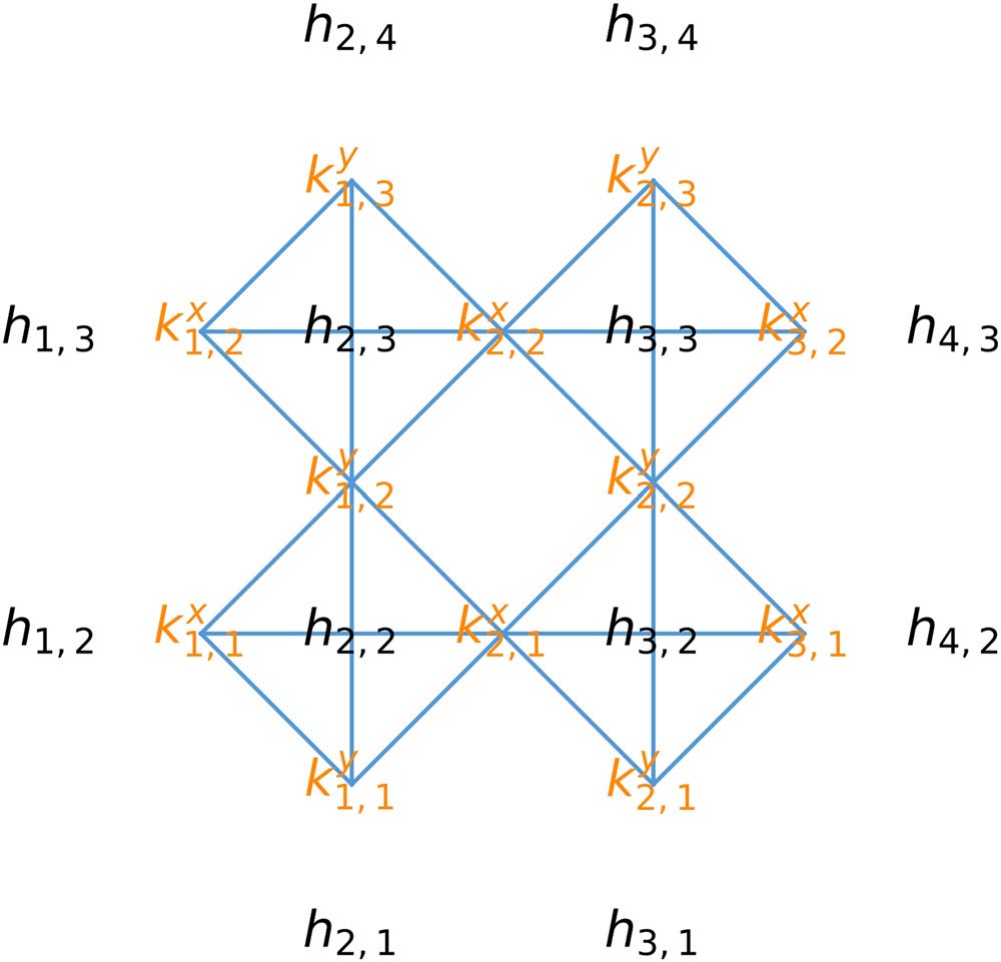
A small-scale 2D finite difference grid is shown with the heads (black), permeabilities (orange), and the couplings between the permeabilities (blue lines).

In two dimensions, we use the following finite difference approximation at head node (*i*, *j*) (assuming Δ*x* = Δ*y* = 1)

3 $$0={k}_{i-\mathrm{1,}j-1}^{x}({h}_{i-\mathrm{1,}j}-{h}_{i,j})+{k}_{i,j-1}^{x}({h}_{i+\mathrm{1,}j}-{h}_{i,j})+{k}_{i-\mathrm{1,}j-1}^{y}({h}_{i,j-1}-{h}_{i,j})+{k}_{i-\mathrm{1,}j}^{y}({h}_{i,j+1}-{h}_{i,j})$$

where ${k}_{i,j}^{x}$ is the anisotropic permeability in the *x* coordinate direction and ${k}_{i,j}^{y}$ is the anisotropic permeability in the *y* coordinate direction. Again, squaring the right hand side of Eq. 3, making a binary approximation of ${k}_{i,j}^{x}$ and ${k}_{i,j}^{y}$, summing over *i* and *j*, and neglecting a constant term, we obtain an objective function similar to Eq. 2. The most important difference from the 1D case is that there are more couplings. Figure 1 visualizes the couplings in a small scale 2D problem–the 2D problem we analyze has a similar structure but utilizes a larger grid. Permeabilities on interior nodes in the 1D problem are coupled to 2 neighboring permeabilities, while they are coupled to 6 neighboring permeabilities in the 2D case.

### Programming the D-Wave

There are two fundamental problems when programming the D-Wave to solve a hydrologic inverse problem. The first is to find the linear and quadratic coefficients in the objective function, $f({\boldsymbol{q}})$ (e.g., Eq. 2). The second is to find a way to transform (using certain rules) a graph associated with the D-Wave system into a graph associated with the hydrologic inverse problem. In this context, a graph does not refer to a visual diagram showing the relationship between quantities, but instead to a collection of vertices connected by edges (i.e., graph theory)^20^. The operations that can be used to transform the graph are edge deletions and contractions. Deleting an edge simply means removing the edge from the graph. Contracting an edge means that the two vertices which are connected by the edge are merged into a single vertex. The edges associated with the new, merged vertex connect it to the vertices that are connected to either (or both) of the two original vertices. This process of transforming the graph associated with the D-Wave into the graph associated with the problem that is being solved is called “embedding.”

The objective function and the embedding transformation can be thought of as inputs to the D-Wave system. The transformation is used to define a new objective function, ${f}^{\ast }({\bf{q}})$ that “fits” on the D-Wave system. Settings^21^ can be tuned in an attempt to make it so that the minima of $f({\bf{q}})$ are also minima (or nearly minima) of ${f}^{\ast }({\bf{q}})$. The behavior of the D-Wave system is stochastic and its output is a set of samples, **q**, that tend to make ${f}^{\ast }({\bf{q}})$ small. To a first approximation, the D-Wave system samples from a Boltzmann distribution. That is, the likelihood of obtaining a sample **q** is proportional to ${e}^{-\beta {f}^{\ast }({\bf{q}})}$ for some *β* which is generally not known *a priori*. Experience indicates that *β* can range from ∼4 for complex problems to ∼17 for simple problems on the D-Wave 2X used in this study. When solving a hydrologic inverse problem, the idea is that one can find the minima (or near minima) of $f({\boldsymbol{q}})$ by repeatedly obtaining samples from the D-Wave and taking the best sample as the solution to the inverse problem.

**Figure 2:**
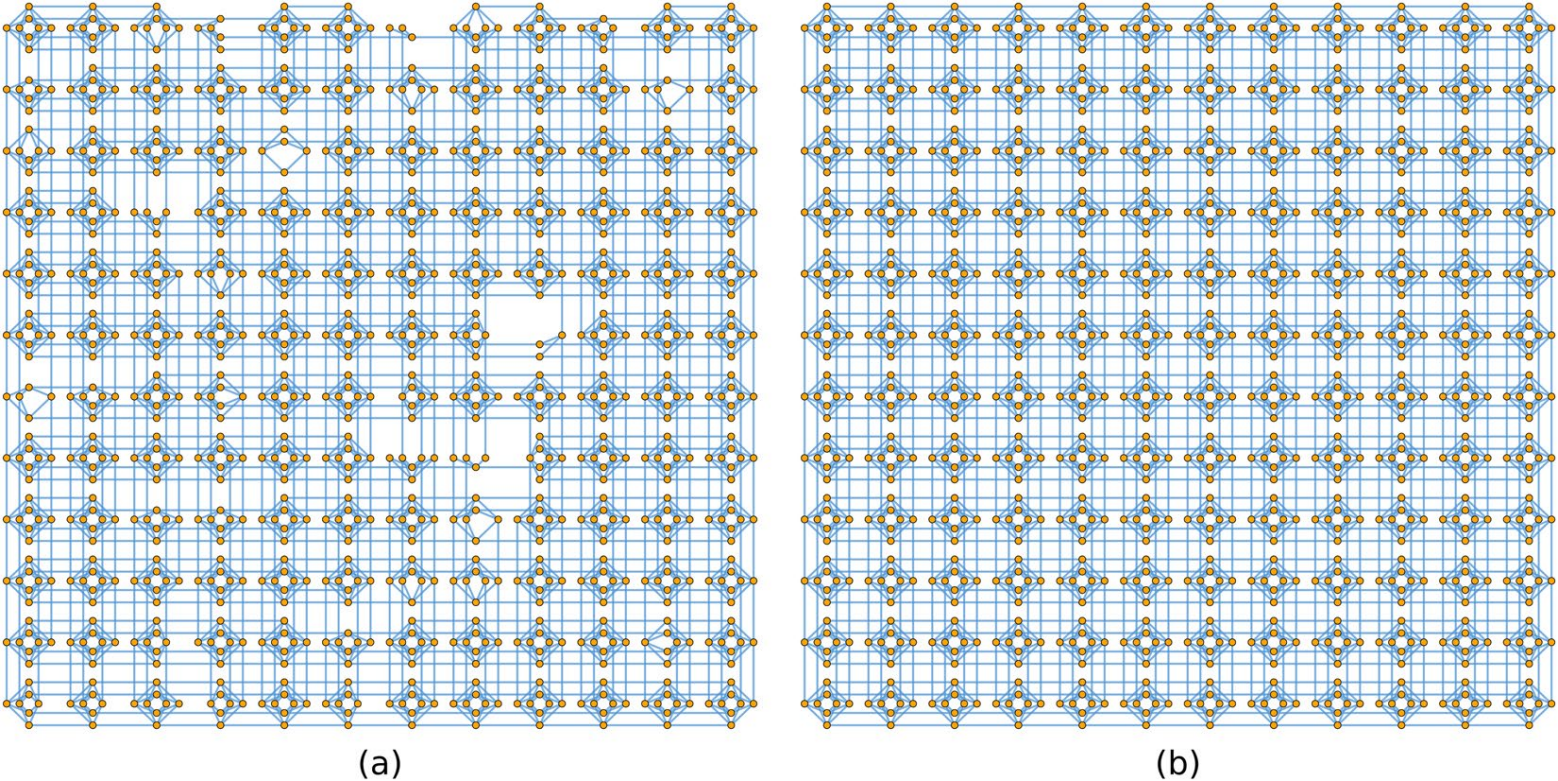
(**a**) The physical hardware graph associated with the D-Wave 2X at Los Alamos National Laboratory and (**b**) the associated Chimera graph are shown. Each vertex in the graph represents a bit and each edge in the graph indicates that the two associated bits can be coupled. Note that (a) is obtained from (b) by removing some vertices and edges.

The problem of finding the coefficients in $f({\bf{q}})$ is very similar to assembling matrices as part of numerical methods (such as finite difference). Since this type of process is familiar to most computational scientists, the details will not be discussed further. The second problem of finding the embedding transformation is less familiar and merits further discussion. The graph associated with the hydrologic inverse problem is formed by adding a vertex for each permeability and adding an edge between two vertices if the corresponding permeabilities are coupled. There are two graphs associated with the D-Wave system, and the user can choose between them. One graph is called the Chimera graph^22^ and is associated with the hardware design. The other, which we call the physical hardware graph, is a subgraph of the Chimera graph that results from some parts of the hardware being inoperable due to imperfections in the production and hardware calibration process. Figure 2 shows both the Chimera graph and the physical hardware graph. When the full Chimera graph is used, the D-Wave system first samples from the physical hardware graph then uses classical post-processing to compensate for the inoperable parts of the hardware.

**Figure 3:**
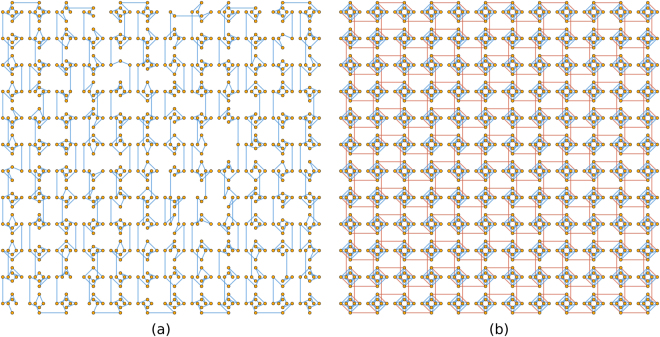
The embeddings associated with the (**a**) 1D and (**b**) 2D inverse problems are shown. The 1D embedding is obtained by removing vertices and edges from the physical hardware graph in Fig. 2a. The 2D embedding is obtained by contracting edges (in particular, the red edges) and removing edges from the Chimera graph in Fig. 2b.

We use the physical hardware graph when solving the 1D hydrologic inverse problem (see Fig. 2a), and the Chimera graph when solving the 2D problem (see Fig. 2b). The graph associated with the 1D problem consists of a sequence of vertices where each vertex is connected only to the vertices coming before and after it in the sequence. The physical hardware graph can be transformed into such a graph by finding a simple path (i.e., one where no vertex is visited more than once) through the graph and removing the edges and vertices that are not part of the simple path. The problem of finding the longest simple path through a large graph is generally computationally intractable^23^. Tools, such as KaLP^24^, are available for finding the longest simple path through graphs, but we found that finding the longest path in the physical hardware graph was computationally intractable using this tool. Finding the longest path may be intractable, but merely finding a long path is much easier. We exploit the structure of the graph (which consists of a 12-by-12 grid of “unit cells” containing at most 8 vertices each), and decompose it into segments in a snake-like pattern where each segment consists of two neighboring unit cells. The KaLP^24^ software is then used to find the longest simple path through each segment, and paths within the segments are connected to form a long path through the entire graph. Figure 3a shows the path found through the graph using this method.

Finding the embedding for the 1D problem required the use of heuristic software to find long paths in a graph, because the graph lacked enough structure to find the embedding without these heuristics. The Chimera graph that is used to solve the 2D problem is more structured, and we can find the embedding more readily. The Chimera graph is comprised of a 12-by-12 grid of unit cells where each cell contains 8 vertices. Each of these unit cells contains two groups (call them group A and group B) of vertices with 4 vertices in each group, and there is an edge between any vertex in group A and any vertex in group B, but no edges connecting vertices within group A, nor any edges connecting vertices within group B (graphs that have these properties are called bipartite graphs). The first step in the transformation is to convert each of the 8-vertex bipartite graphs that comprise a unit cell into a 4 vertex complete graph. A complete graph is one where there is an edge between any two vertices. A complete graph with 4 vertices can be obtained from the 8-vertex bipartite graph in each unit cell by associating each vertex in group A with a vertex in group B and contracting the edge connecting them. These contractions are depicted by the diagonal red lines in Fig. 3b. This first step essentially produces a 12-by-12 grid of 4-vertex complete graphs where there are also some edges connecting vertices in each of the 4-vertex complete graphs with vertices in neighboring 4-vertex complete graphs. Notice that there are four complete graphs with 4 vertices each (here we think of the permeabilities as being vertices) in Fig. 1 arranged in a 2-by-2 grid. The 12-by-12 grid of unit cells in the Chimera graph is transformed into a 12-by-12 grid similar to the 2-by-2 grid in Fig. 1. For this transformation to work, it must be recognized that each vertex in the graph from Fig. 1 belongs to two 4-vertex complete graphs. Respecting this fact requires additional edge contractions represented by the horizontal and vertical red lines in Fig. 3b. The Supporting Information contains a video that visualizes the transformation from the Chimera graph in Fig. 2b to a 12-by-12 grid similar to the one in Fig. 1.

## Results

We describe results from three examples. The first is a very small 1D example that is included to explicitly demonstrate some of the methods. The second is a larger 1D example, and the third is a 2D example. In all of the examples, the goal is to segment the aquifer into regions of low permeability, *k*_*l*_, and high permeability, *k*_*h*_. This would be an appropriate approach when the aquifer has a bimodal permeability distribution, and the goal is to associate the permeability at each location with one of the modes. This decomposition of the permeability field into high and low permeability regions is essentially the same as in level set methods^19^ that are used on classical computers. For notation, we will use *h*_*i*_ to denote the hydraulic head at node *i*, *k*_*i*_ to denote the permeability in 1D, and ${k}_{i,j}^{x}$ (${k}_{i,j}^{y}$) to denote the horizontal (vertical) permeability in 2D. We also denote the hydraulic head observations with ${\hat{h}}_{i}$ (which are the input to the inverse analysis) and the inverted permeabilities with ${\hat{k}}_{i}$ (which are the output of the inverse analysis).

### Small 1D problem

Consider a finite difference discretization of the groundwater flow problem with 3 head nodes, where *h*_1_, *h*_2_, and *h*_3_ represent the hydraulic head at *x* = 0, 1, and 2, respectively. Suppose that it is known that *h*_1_ = 1, *h*_2_ = 1/3 and *h*_3_ = 0. These are the values of *h*_*i*_ that would be obtained if *k*_1_ = 1 and *k*_2_ = 2, so the goal of our inverse problem is to obtain these values. Using Eq. 1, the finite difference approximation about *h*_2_ is 0 = *k*_1_(*h*_1_ − *h*_2_) + *k*_2_(*h*_3_ − *h*_2_). We first modify this equation by inserting the known values for *h*_1_, *h*_2_, and *h*_3_ to obtain 0 = 2*k*_1_/3 − *k*_2_/3. The D-Wave system cannot solve equations *per se*, but it can solve least-squares problem; so we reformulate this equation as a least squares problem. That is we seek *k*_1_ and *k*_2_ that minimize the objective function *f*(*k*_1_, *k*_2_) = (2*k*_1_/3 − *k*_2_/3)^2^. As mentioned previously, we discretize the permeabilities as *k*_*i*_ = *k*_*l*_ + *q*_*i*_(*k*_*h*_ − *k*_*l*_) where *q*_*i*_ is a binary variable that we solve for with the D-Wave system. Here we use *k*_*l*_ = 1 and *k*_*h*_ = 2, so the correct inverse solution in terms of the binary variables is *q*_1_ = 0 and *q*_2_ = 1. Reformulating the objective function in terms of *q*_*i*_, we obtain *f*(*q*_1_, *q*_2_) = 8*q*_1_/9 − *q*_2_/9 − 4*q*_1_*q*_2_/9 + 1/9 where we have used the fact that ${q}_{i}^{2}={q}_{i}$ (since *q*_*i*_ is binary) to simplify this expression and the 1/9 term can be ignored.

Finding the embedding for this problem is simple, since there are only two binary variables. We only need to find a subgraph of the graph in Fig. 2a with two vertices that are connected by an edge, and such subgraphs are abundant. We submitted this problem to the D-Wave and obtained 1,000,000 samples of **q** = (*q*_1_, *q*_2_). The correct solution of **q** = (0, 1) was obtained with probability ∼0.863. This means that the expected annealing time required to obtain the correct solution is around 23 *μs*, since the annealing time to obtain a single sample was 20 *μs*. The second most likely outcome was **q** = (0, 0) which was obtained with probability ∼0.136. The other outcomes (**q** = (1, 0) and **q** = (1, 1)) were each obtained with probability less than 0.001. These probabilities are in good agreement with the probabilities obtained from the Boltzmann likelihood *e*^−*βf*(**q**)^ with *β* ≈ 16.6. That is, the D-Wave acts as a sampling device where the samples are taken (to a first approximation) from a Boltzmann distribution where $f({\bf{q}})$ acts as the energy.

### Larger 1D problem

On a D-Wave 2X chip with 1095 operational bits, we were able to solve 1D inverse problems with 972 permeabilities using the embedding shown in Fig. 3a. Viewing the problem through a needle-and-a-haystack analogy, there are $\sim {10}^{293}$ (i.e., 2^972^ − 1) pieces of hay (i.e., incorrect solutions) and one needle (i.e., one correct solution) for this set of problems. We performed a series of analyses where we varied the observational noise level, *σ*, between 10^−6^ and 10^−2^, and *k*_*h*_ between 10^1/10^ and 10^1/2^, while *k*_*l*_ was fixed at 1. In each analysis, we generated 100 different realizations of the permeabilities, *k*_*i*_, where each *k*_*i*_ has equal probability of being either *k*_*l*_ or *k*_*h*_. This realization of the permeability field was used to obtain values of the hydraulic heads, *h*_*i*_, by solving the forward model. Finally, noise was added to obtain ${\hat{h}}_{i}\,={h}_{i}+\sigma Z$ where *Z* is a standard normal random variable–that is we added Gaussian noise with standard deviation *σ*. For each realization, we obtained 10,000 samples from the D-Wave system using the head observations, ${\hat{h}}_{i}$, to invert for permeabilities, ${\hat{k}}_{i}$.

**Figure 4:**
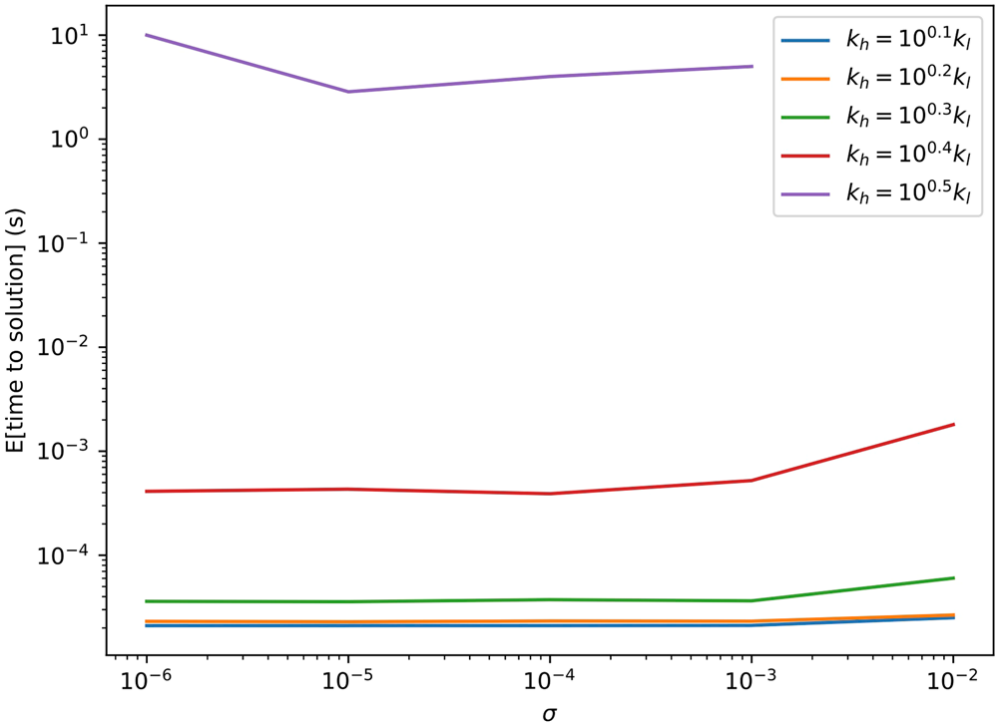
The expected annealing time required to obtain the correct permeabilities on the 1D inverse problems with 972 permeabilities using the D-Wave 2X from the observed hydraulic heads is shown as a function of the noise level (*x*-axis) and permeability contrast (the different colored curves).

Figure 4 shows the expected amount of annealing time required to obtain the the correct inverse solution (i.e., ${\hat{k}}_{i}$ = ${k}_{i}$ for every *i* = 1, 2, …, 972). Some general trends can be readily observed. First, increasing the noise, *σ*, from 10^−3^ to 10^−2^ increases the time required to obtain the correct inverse solution, but the difference in solution time at lower noise levels is negligible. This trend is to be expected–sufficiently noisy data makes inverse analysis more difficult. Second, and perhaps more surprising, is that as the contrast in permeability between *k*_*h*_ and *k*_*l*_ increases, the D-Wave system requires more time to obtain the correct solution. This is an important limitation, because permeability often varies by several orders of magnitudes in real aquifers. This difficulty in dealing with large contrasts in permeability could be mitigated by improvements in the quantum annealing hardware (such as lowering the temperature or improving the precision with which the coefficients in Eq. 2 can be controlled), or potentially with improvements on the methods presented here.

### 2D problem

We now consider a 2D problem that exploits the Chimera graph in Fig. 2b via the embedding in Fig. 3b. Similar to the larger 1D problem, we generated random permeability fields where each permeability in the fields had permeability of either *k*_*l*_ or *k*_*h*_ with equal probability. For this problem, *k*_*h*_ was taken to be 2 and *k*_*l*_ was taken to be 1 in all tests. These fields were used to obtain simulated hydraulic heads that were treated as observations for the purpose of the inverse analysis (no noise was added here). In this case, there are essentially two permeability fields for each realization. One field describes the anisotropic permeability aligned in the *x*-axis, ${k}_{i,j}^{x}$, on a 13-by-12 grid, and another describes the anisotropic permeability aligned with the *y*-axis, ${k}_{i,j}^{y}$ on a 12-by-13 grid.

**Figure 5:**
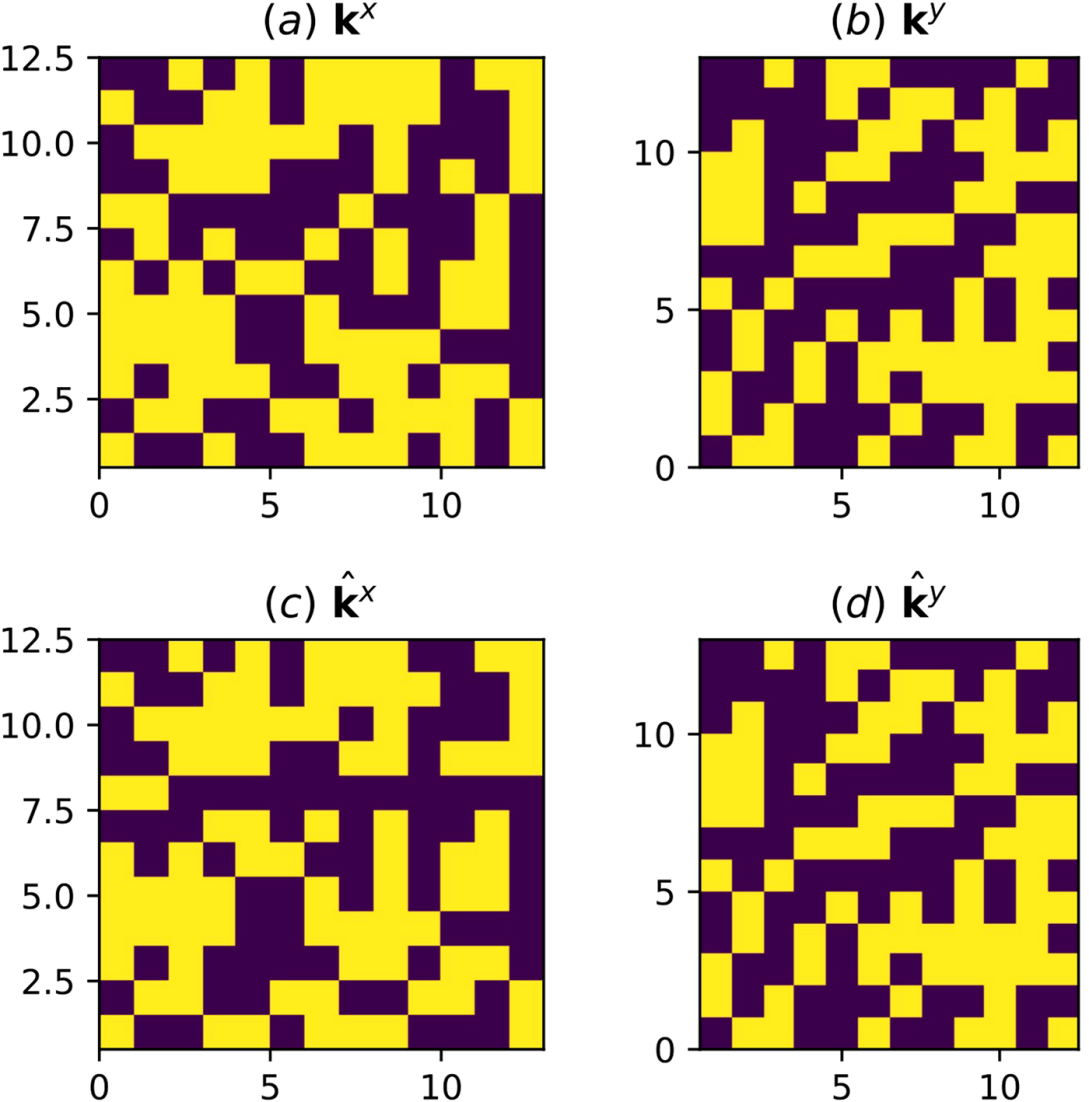
The (**a**) horizontal and (**b**) vertical permeability fields used to generate the hydraulic head observations are shown as is the best inverse result for the (**c**) horizontal and (**d**) vertical permeability. The purple regions have permeability *k*_*l*_ and the yellow regions have permeability *k*_*h*_. Note that ${\hat{{\bf{k}}}}^{{y}}={{\bf{k}}}^{{y}}$, while ${\hat{{\bf{k}}}}^{{x}}={{\bf{k}}}^{x}$ differ in some locations.

**Figure 6:**
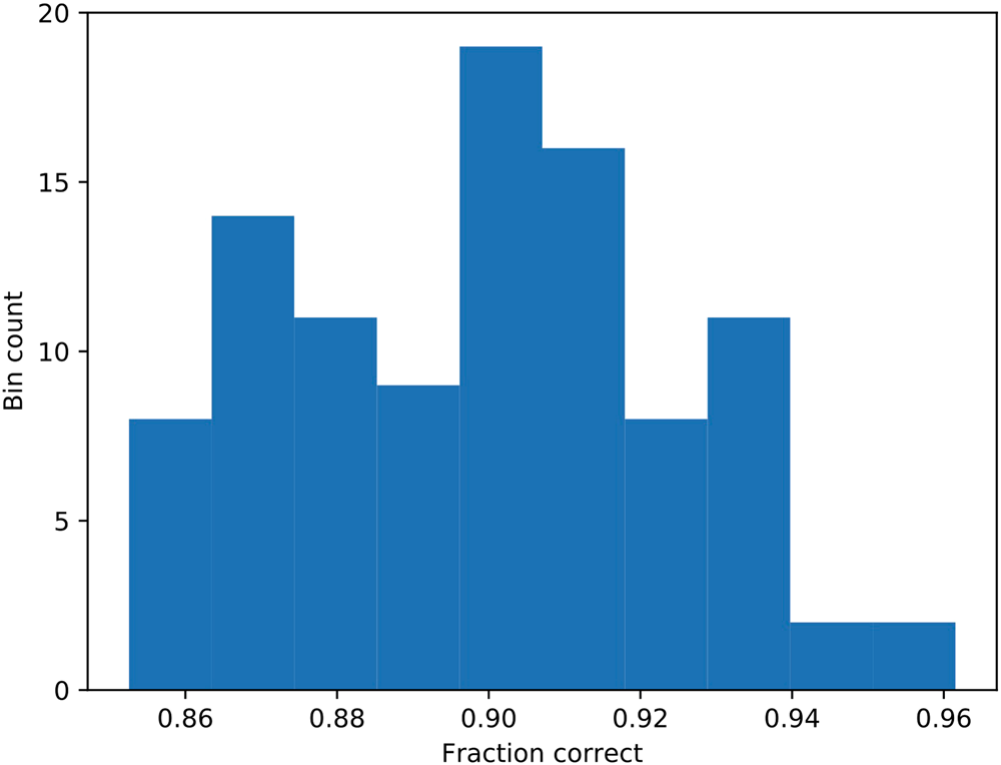
A histogram of the fraction of ${k}_{i,j}^{x}$ values that were correctly obtained in the 2D inverse problem is shown. Note that in all 100 cases, all of the ${k}_{i,j}^{y}$ values were correctly obtained.

The 2D inverse analysis has a low probability of inverting for the correct permeabilities given the hydraulic head observations, even with no noise in the observations. This is primarily because the D-Wave system has difficulty obtaining the correct ${k}_{i,j}^{x}$, rather than ${k}_{i,j}^{y}$. Note that in our tests, the gradient in the hydraulic head was aligned with the *y* axis, producing small gradients in the *x* direction that made the inverse analysis less sensitive to ${k}_{i,j}^{x}$ than to ${k}_{i,j}^{y}$. While ${k}_{i,j}^{x}$ was not inverted for exactly in our tests, there is a reasonably high probability of obtaining ${k}_{i,j}^{y}$ exactly and a fairly good approximation of ${k}_{i,j}^{x}$. For example, Fig. 5 shows the best result from an inverse analysis for the 2D problem where ${k}_{i,j}^{y}$ was obtained exactly and ${k}_{i,j}^{x}$ was obtained approximately. We performed 100 inverse analyses for the 2D problem. For each inverse analysis, 10,000 samples were obtained from the D-Wave. In all 100 cases, the best result obtained ${k}_{i,j}^{y}$ exactly, but none obtained ${k}_{i,j}^{x}$ exactly. Figure 6 shows the histogram of the fraction of the ${k}_{i,j}^{x}$ that were correct with the median value of this fraction being 90%. Even though the D-Wave was unable to obtain the exact correct result with 10,000 samples, it was able to obtain a good result in all cases–${k}_{i,j}^{y}$ was correct in all locations and ${k}_{i,j}^{x}$ was correct in 85% of locations (in the worst case). Obtaining 10,000 samples may seem like a lot, but it requires a modest computational investment amounting to 0.2*s* of annealing time (each anneal takes 20 *μs*).

It is natural to wonder how the performance of quantum annealing compares to classical computing for these problems. The answer depends on what classical algorithm and computer is being used as well as how the comparison is performed. How to perform these comparisons remains an active area of research^25–29^. We performed a simple benchmark to compare the performance of the D-Wave 2X system to a classical algorithm for the 2D hydrologic inverse problems. The algorithm is implemented in a commercial piece of software called Gurobi^30^ (a state-of-the-art mathematical programming solver). The comparison utilized a version of the time-to-target benchmark^28^ where we attempt to find how much time Gurobi requires to obtain a solution that is as good as or better than the best solution (among the 10,000 samples) obtained by the D-Wave 2X for each of the 100 realizations of the 2D inverse problem. We call this time the time-to-target. The classical computer used for the comparison had 256 gigabytes of memory and 2 Intel Xeon E5-2699 CPUs running at 2.2 GHz with 22 cores and 44 threads each for a total of 88 logical cores. Gurobi was run in parallel using 88 threads, and Gurobi was given a time limit of 15 minutes of wall time per problem.

We group the 100 inverse problems into three categories. The first category contains problems that were relatively easy for Gurobi, where the time-to-target was less than 10 seconds. The second category contains problems of moderate difficulty for Gurobi, where the time-to-target was between 10 seconds and 15 minutes. The third category contains problems where the time-to-target exceeded 15 minutes. There were 28 problems in the first category, 8 problems in the second category, and 64 problems in the third category. This indicates that finding a solution that is at least as good as the D-Wave’s best solution is difficult for Gurobi in most cases even when equipped with a relatively fast classical computer and the D-Wave obtains only 10,000 samples. In an effort to estimate how long it would take for Gurobi to match the D-Wave’s best solution for problems in the third category, we removed the wall time restriction for one problem in the third category. For this problem, the classical computer ran out of memory after about 4 hours of wall time. We reran the problem again using a flag recommended by Gurobi’s documentation for problems that run out of memory, and, when this flag was used, Gurobi ran for 24 hours without finding a solution that is as good as or better than the D-Wave’s best solution.

It is important to put this comparison in proper perspective. Gurobi was used here because it makes for an interesting historical comparison with Hefez *et al*.^2^ where the lack of an off-the-shelf or “readily available computer program” for solving quadratic programming problems was bemoaned. We emphasize again that performance is highly dependent on the classical algorithm that is used, and we expect that other classical algorithms would outperform the D-Wave on these problems. Algorithms such as the Hamze-de Freitas-Selby algorithm^31^ or a highly optimized simulated annealing algorithm^32^ would be good candidates for potentially outperforming the D-Wave on these problems. We leave exploration of alternative algorithms for future work–our intention is to make a historical comparison to the work of Hefez *et al*.^2^. Using methods similar to Hefez *et al*.^2^, the D-Wave 2X outperformed a relatively fast classical computer using Gurobi (which we regard as the modern version of the “readily available computer program” for solving quadratic programs that was not available during the time of Hefez *et al*.). From this historical perspective, these results are promising for quantum annealers. However, it remains to be seen if improvements in quantum annealers in the years to come will mirror those of classical computers in years past. Improvements of this nature are likely needed before practical applications of quantum annealing to hydrology arise.

## Discussion

While quantum computing technology is in an early stage, our intention is to demonstrate that it has progressed to the point where proof-of-principle calculations can be performed for certain subsurface flow problems. Progress must be made on multiple fronts before quantum computing is ready for practical applications to these sorts of problems. Improvements in the hardware will likely be a major driver of this progress, but methodological improvements should also be pursued. Direct inverse methods have fallen out of favor, because hydraulic head observations are typically sparsely distributed in an aquifer. Indirect methods that utilize quantum annealing can be developed. For example, an iterative method that relies on discrete quadratic approximations of the objective function (similar to Newton’s method for optimization) could be used that does not require dense observations of the hydraulic head.

In addition to the approach to inverse analysis demonstrated here, quantum annealing also holds promise for performing uncertainty quantification on these subsurface flow problems. We have carried out preliminary analyses in this direction that utilize the Boltzmann approximation (i.e., treating the samples from the D-Wave as samples from a Boltzmann distribution). While the Boltzmann approximation performed fairly well in the small 1D problem that we studied, the approximation was not sufficiently accurate to carry out importance sampling on larger problems. Further work that improves on the Boltzmann approximation of the D-Wave’s sampling behavior or uses alternatives to the importance sampling could enable quantum annealing to accelerate hydrologic uncertainty quantification problems.

The proof-of-principle computations performed here show some promise for the use of quantum annealing in subsurface hydrology. It is worth noting that, while possible to solve the forward problem (i.e., solving for the hydraulic head given the permeability) with quantum annealing^33^, it is more natural to solve the inverse problem. The opposite is generally true for classical computers–it is more natural to solve the forward problem than the inverse problem. Further, the discrete nature of the inverse problems solved here (where the permeability is either *k*_*l*_ or *k*_*h*_ and cannot vary continuously) make them suitable for quantum annealing, but very difficult for classical computers^34^. Recent work in hydrologic inverse analysis has enabled the efficient solution of large-scale problems where the permeabilities are treated continuously (often as a multivariate Gaussian)^35,36^. This continuous problem can be seen as inferring the variability within a hydrologic unit while the binary problem solved here can be used to differentiate one hydrologic unit from another. We take this as an encouraging sign that quantum annealers and classical computers are well-suited to solve a complementary set of problems.

## Electronic supplementary material

Supplementary Information
